# Nanosilver Biocidal Properties and Their Application in Disinfection of Hatchers in Poultry Processing Plants

**DOI:** 10.1155/2016/5214783

**Published:** 2016-01-24

**Authors:** Marcin Banach, Leszek Tymczyna, Anna Chmielowiec-Korzeniowska, Jolanta Pulit-Prociak

**Affiliations:** ^1^Cracow University of Technology, Warszawska 24, 31-155 Kraków, Poland; ^2^University of Life Sciences, Akademicka 13, 20-950 Lublin, Poland

## Abstract

The aim of this study was to use aqueous suspensions of silver nanoparticles with a wide spectrum of particle sizes, variable morphology, high stability, and appropriate physicochemical properties to examine their bactericidal and fungicidal properties against microorganisms present in poultry processing plants. At the same time, the particles were tested for preventing the production of odorogenous pollutants during incubation and thereby reducing the emission of harmful gases from such types of facilities. The results show that the use of nanosilver preparations in order to disinfect eggs and hatchers reduced microbiological contamination. The bactericidal and fungicidal efficacy of the applied preparation was comparable to UV radiation and its effectiveness increasing during the incubation. Good results were achieved in terms of the level of organic gaseous contaminants, which decreased by 86% after the application of the nanosilver preparation.

## 1. Introduction

Poultry farming and related chick hatching processes, which base their production technology on biological material, are a potential source of microbiological contamination, including microorganisms and their toxins. These factors are one of the components of bioaerosols, particularly organic dust. They are shunted outside through a ventilation system and remain at high levels up to one kilometre from the source of emission. Many poultry diseases, including respiratory tract infections, are transmitted through the air over long distances, even reaching locations three kilometres away from the place of formation [1–8].

Research conducted by Skórska et al. [9] at poultry processing plants in the 1990s showed high levels of contamination in the hatchery hall. The mean concentration of microorganisms in the air exceeded 37,000 cfu/m^3^ and increased up to 310,000 cfu/m^3^ when the chicks were being taken out. Nowadays, even the new technologies introduced to modernised plants according to the requirements of the European Union do not totally eliminate biological air pollution. Research by Tymczyna et al. [1–6] demonstrated that the mean bacteria concentration in the air of a hatchery hall amounted to 4,000 cfu/m^3^. In the bioaerosol from the hatchery hall, a relatively high proportion (16%) of Gram-negative bacteria was observed, and the following genera of bacteria were identified:* Acinetobacter*,* Citrobacter*,* Enterobacter*,* Escherichia*,* Flavobacterium*,* Klebsiella*,* Pseudomonas*,* Leclercia*,* Sphingomonas*,* Xanthomonas, Agrobacterium*, and* Pantoea*.

In chick hatcheries, Gram-negative rods of* Acinetobacter calcoaceticus* constitute about 40% of all bacterial strains and are one of the most significant sources of endotoxins in the air.* Alcaligenes faecalis*,* Erwinia herbicola*,* Enterobacter *spp., and* Pseudomonas *spp. are other sources of endotoxins in the air. Under certain circumstances, some species of Gram-negative bacteria capable of causing infectious diseases, such as* Klebsiella pneumoniae* which causes pneumonitis or rods of the* Salmonella* genus which are the cause of salmonellosis, can penetrate into the air. Another microorganism that was observed in chick hatcheries is* Alcaligenes faecalis*, which exhibits endotoxic and allergenic properties.

Particularly noteworthy are Gram-negative bacteria of* Erwinia herbicola* (synonym:* Pantoea agglomerans *and* Enterobacter agglomerans*), which are a source of potent allergens and endotoxins. Bacteria of the* Pantoea* genus are the most numerous Gram-negative microflora in the aerogenic dust of plant origin. Gram-negative rods* Alcaligenes faecalis* and* Erwinia herbicola, *due to their ability to both specifically and nonspecifically activate the immune system, can induce inflammatory states in the lungs and cause respiratory system diseases (organic dust toxic syndrome) [1–8].

In research conducted by Stępień-Pyśniak et al. [10], among the most frequently isolated bacteria of the family,* Enterobacteriaceae, Escherichia coli*,* Enterobacter *spp.,* Klebsiella *spp., and* Citrobacter freundii* were identified. On the surface of eggshells, the presence of* Salmonella* rods, that is,* S. enteritidis* and* S. arizonae*, has been observed. The qualitative analysis of bacterial microflora of the eggs revealed also the presence of the other Gram-negative bacteria:* Acinetobacter *spp.,* Pseudomonas *spp.,* Tatumella ptyseos*,* Providencia stuartii*,* Serratia liquefaciens*,* Flavimonas oryzihabitans*,* Vibrio metschnikovii*,* Leclercia adecarboxylata*,* Kluyvera *spp.,* Rahnella aquatilis*,* Proteus mirabilis*, and* Achromobacter *spp. Moreover, it has been shown that a high proportion of examined eggshells were contaminated with bacteria of the genus* Staphylococcus *spp. Apart from staphylococci,* Enterococcus *spp. and rods of the genus* Bacillus* are often isolated from the surface of the eggshells. Microbiological contamination of eggshells can be a cause of contamination of egg products or products containing eggs and, as a consequence, can lead to intoxication or food infections in people. This is mainly a result of failure to comply with hygiene standards. Hence, the microbiological purity of eggshells is one of the main criteria in assessing the value of this product in marketing and processing [10, 11].

According to the present standards of production, in the majority of food processing plants, including the egg and poultry industry, obligatory control of microbiological and chemical quality of the air and production area, as well as the obligation to ensure proper purity, has been introduced. The system for assessing critical control points in production on the basis of hazard analysis (Hazard Analysis Critical Control Points, HACCP), which has been introduced in the food industry, ensures microbiological safety of the product and provides effective protection against reinfection. This method allows for localising the places and sources of risk for biological, physical, and chemical hazards at particular stages of production and also enables plants to set critical control points where these hazards can be controlled, monitored, and eliminated (or minimalized). In poultry hatchery plants, these critical control points are the hatchers, where thermal and humidity conditions and the presence of biological material pose a significant microbiological hazard. As a result of intensive changes inside the incubators, a wide range of gaseous compounds accumulate, which are released through the micropores in the eggshells as a result of microbiological transformations on the surface, as well as originating from disinfecting agents. In such places, not without reason, great emphasis is put on maintaining microbiological purity, which is achieved only by properly conducted disinfection of hatchers and eggs [1–8].

In addition to microbiological contaminants of poultry production, a variety of chemical substances originate as a result of metabolic changes in developing embryos. The gaseous environment of the embryo is an extremely important factor in the process of incubation, especially during the first 96 hours. Between the 38th and the 48th hours of embryogenesis, allantois growth occurs, the vessel field develops on the umbilical vesicle, and all the organs start to develop. If any of the organs fails to develop as a result of metabolic disorders or the influence of toxic substances, lethal disturbances in foetal circulation occur, leading to clot formation in rete vasculosa. Up to the 8th day of incubation, the umbilical vesicle and the allantoic vessels play the role of a respiratory organ. The conversion to pulmonary respiration occurs slowly, during the retraction of the umbilical vesicle during the final stage of incubation. During this period, gaseous compounds can cross the eggshell barrier in both directions, causing developmental disorders and embryo decay [1–8].

In the air of the chick hatchery, apart from ammonia, hydrogen sulphide, mercaptans, aldehydes, and ketones, high concentrations of acrolein, acetaldehyde, and predominantly formaldehyde, which is mainly used for the disinfection of rooms, have been detected. Other gases, such as m- and p-tolualdehyde, benzaldehyde, and isovaleric and butyl aldehyde, are present in rather trace amounts. It has been demonstrated that 1 m^3^ of air contains on average about 7.7 g of organic gas contamination.

The harmful mode of action of acetaldehyde is demonstrated by its narcotic effect on the central nervous system, irritation of the skin and mucous membranes, reduction in blood pressure, and heart palpitations. Similarly, acrolein is a substance that is irritating to mucous membranes and conjunctiva. It has rapid and direct action on the respiratory tract.

In the hatchery hall, the presence of benzaldehyde, an oily substance with a characteristic smell of bitter almonds, has also been detected. In people, benzaldehyde causes irritation of the mucous membranes and, when inhaled in great amounts through the respiratory tract, induces allergic symptoms and central nervous system disorders and has also been associated with cytostatic and carcinogenic effects. In previously conducted research, it has been shown that concentrations of these selected compounds were lower compared to the TLV (threshold limit value) for air. However, trace amounts of toxic gases are still potentially harmful, because these contaminants can act on organisms independently; they can neutralise their action or increase their mutual harmful effects [1–10].

The number of chemical products used for disinfection in food processing is limited due to the negative impact of these compounds on the human body and because of difficulties with solubility and the possibility of direct application. Moreover, the environmentally friendly lifestyle of many consumers forces food technology to use only chemical compounds that occur in nature. Among the preferred additives that damage bacterial flora, organic acids and their salts, which are generally considered as safe, are a large group. Citric acid belongs to this group and is approved for use in the food industry. Studies concerning the destruction of microorganisms have been conducted using a variety of methods, with the use of a variety of chemical compounds including organic acids, hexadecylpyridinium chloride, sodium orthophosphate, hydrogen peroxide, and sodium bicarbonate. Not all of these methods turned out to be effective, so there is a continuous search for effective methods of destroying bacteria and fungi. Formaldehyde is commonly used in hatchery plants because of its low cost and effective biocidal action, with little attention paid to the toxic and carcinogenic effects of its use [1–10]. This compound has the ability to damage most microorganisms existing on the outer layer of eggshells. It is used in gaseous form or as a 40% water solution known as formalin. Formaldehyde is an easy to use and effective disinfecting agent but causes irritation of the eyes and mucous membranes of the respiratory system. It is a strong protoplasmic poison, inducing degenerative changes to the cells of liver parenchyma, kidneys, and heart. In the eye, the degenerative changes affect not only the retina, but also the optic nerve and the corneal epithelium.

Silver nanoparticles, which according to the literature show good biocidal properties, may be an alternative. The proposed preparations may also be an effective means of neutralising gaseous contaminants produced during the process of incubation [12–14]. Due to its biocidal properties, nanosilver has become an important and valuable commercial product in the food industry (e.g., packaging and food storage containers), the clothing industry (e.g., antibacterial clothes), the medical industry (e.g., gauze dressings), and others [15–17].

Silver nanoparticles are effective damaging factors for a wide range of Gram-negative and Gram-positive bacteria, not excluding strains resistant to antibiotics [18]. Gram-negative bacteria include the genera* Acinetobacter *[19],* Escherichia *[20],* Pseudomonas *[21],* Salmonella*, and* Vibrio *[22], while Gram-positive bacteria include genera such as* Bacillus *[23],* Clostridium *[24],* Enterococcus *[25],* Listeria *[26],* Staphylococcus *[27], and* Streptococcus *[28]. Bacteria resistant to antibiotics are most often resistant to metacycline and vancomycin, including some strains of* Staphylococcus aureus* and* Enterococcus faecium*. Recent research has shown that silver nanoparticles with a diameter of 22.5 nm increase the antibacterial activity of some antibiotics, such as penicillin G, amoxicillin, erythromycin, clindamycin, and vancomycin [29]. More recent research has revealed very promising properties of nanosilver against viruses, even the HIV-1 virus. Sun et al. observed that silver nanoparticles inhibit the replication of this virus [30, 31].

Applying nanosilver to eradicate fungi also gives satisfying results. Studies have confirmed that silver nanoparticles are an effective and rapidly acting factor against a wide variety of common fungi, including genera such as* Aspergillus *[32],* Candida *[33], and* Saccharomyces *[18]. Moreover, it has been shown that silver nanoparticles show marked activity against yeast isolated from infected cow udders [34].

Nanosilver has been used in animal breeding as a disinfecting agent used to sanitise transport chambers or the space used for the storage of animals. Studies have been conducted to determine the level of ammonia emission from sheep manure after applying a preparation based on silver nanoparticles with the addition of a mineral sorbent. It was concluded that the use of the preparation resulted in a reduction in ammonia emissions from the ground [35].

The aim of this study was to develop methods of obtaining aqueous suspensions of silver nanoparticles with a wide spectrum of particle size and morphology, with high stability and appropriate physicochemical properties, as well as examine their bactericidal and fungicidal properties against microorganisms present in poultry processing plants. At the same time, the particles were tested for their ability to prevent the production of odorogenous pollutants during incubation, thereby resulting in a reduction in the emission of harmful gases from this type of facility. Thus, it was possible to prevent odour generation at the source of its production.

## 2. Materials and Methods

### 2.1. Obtaining and Characteristics of Silver Nanoparticles

The process of obtaining nanostructured silver by the chemical reduction of silver ions was investigated. Silver nitrate (V) (POCh, p.a. grade) served as the source of silver ions. In order to reduce Ag^+^ ions, L(+) ascorbic acid (POCh, p.a. grade) or glucose (POCh, p.a. grade) was used, whereas sodium pyrophosphate (POCh, p.a. grade), gelatine (POCh, p.a. grade), or sodium tripolyphosphate (SIGMA ALDRICH, p.a. grade) was responsible for the stabilisation of forming agglomerates of metallic silver. An aqueous solution of NaOH (0.1 M) was used for pH regulation. Application of these substrates enabled a reduction in the use of hazardous substances in the synthesis of nanosilver, making the process environmentally friendly and consistent with the principles of green chemistry.

A PARR 4525 pressure reactor was used in the process. An aqueous solution of the stabiliser (50.0 cm^3^) was added to the aqueous solution of AgNO_3_ (100.0 cm^3^; 0.001 mol/dm^3^). The solution was heated in a reactor up to a temperature of 110–150°C. After obtaining the set temperature, an aqueous solution of the reducer (50.0 cm^3^) was added to the reactor using a pump. The reduction reaction was carried out for 2–30 min. The parameters of the process of obtaining silver nanoparticles are summarised in Table 1.

A spectrophotometric analysis (UV-Vis) of the obtained suspensions of nanoparticles was carried out on a RAYLEIGH UV-1800 spectrophotometer in the wavelength range from 300 to 600 nm with a resolution of 2 nm. Determination of size of the obtained nanoparticles was performed using the dynamic light scattering (DLS) technique with a Zetasizer Nano-ZS particle size analyser. This device was additionally used to investigate the value of the electrokinetic potential (*ζ*). The obtained silver nanoparticles were visualised using atomic force microscopy (AFM) on a NanoScope V device (Veeco Company, USA). The powder form of the product, obtained by centrifugation (90000 rpm) using a Thermo Scientific Sorvall MX 150 microultracentrifuge with an S140A rotor, was characterised with the use of scanning electron microscopy, performed on a 1430 VP LEO device (Electron Microscopy Ltd.).

**Figure 1:**
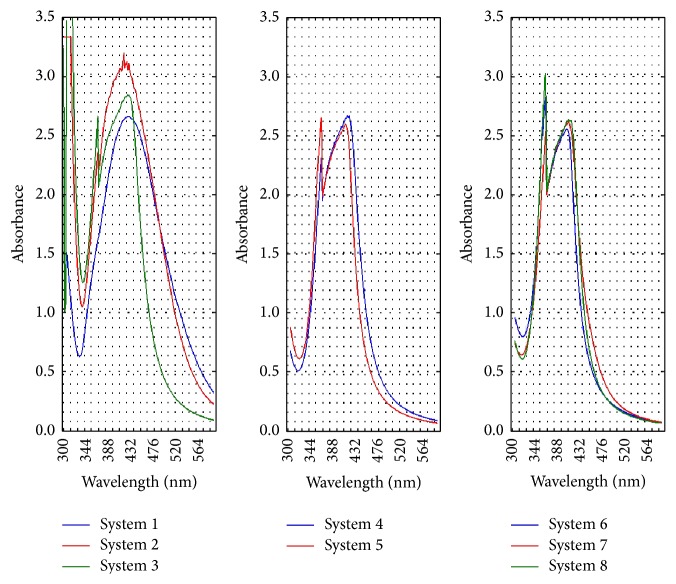
The absorption spectra obtained for the respective suspensions of silver nanoparticles (diluted to 50 mg/dm^3^).

The UV-Vis absorption spectra of the obtained nanosilver suspensions are shown in Figure 1. The peak at 400–450 nm corresponds to the characteristic surface plasmon resonance of silver nanoparticles. Surface plasmons are coherent oscillations of valence electrons of the atoms present on the surface of a material.

**Figure 2:**
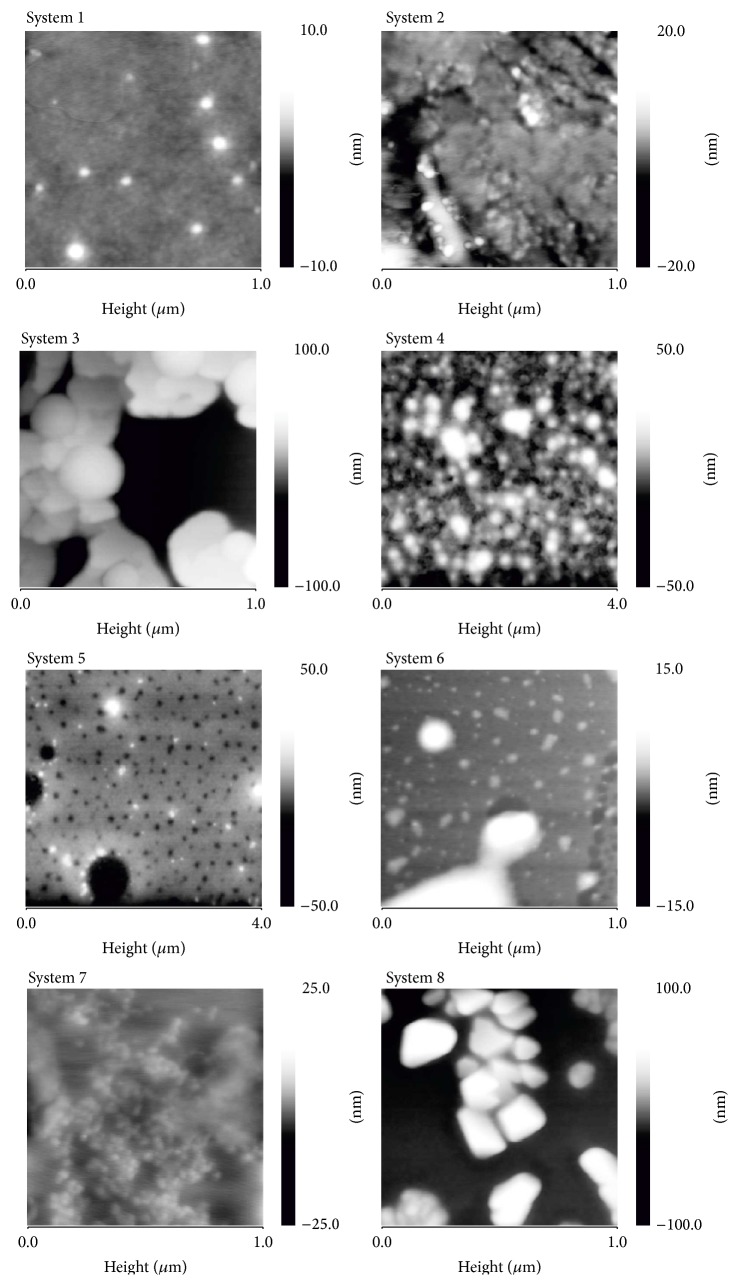
AFM photographs of the silver nanoparticles.

**Figure 3:**
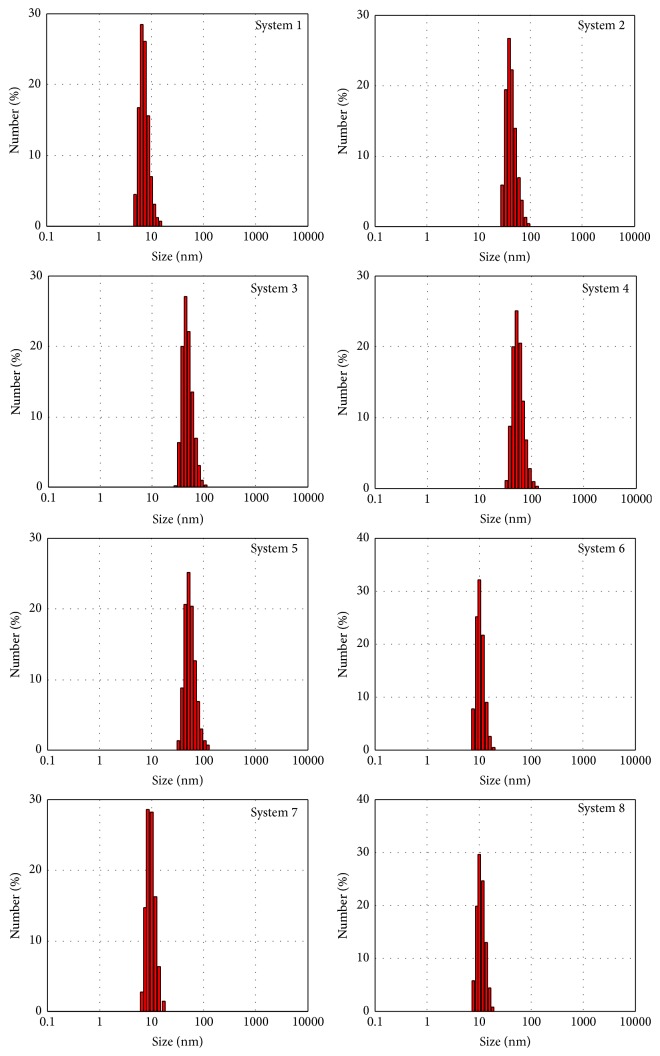
The size distribution of silver nanoparticles.

Absorption of radiation by metallic nanoparticles mainly depends on their size and shape. Plasmon assembly was not symmetric, which means that the solutions contained aggregated particles. This was confirmed by the atomic force microscopy photographs shown in Figure 2. The obtained UV-Vis absorption bands were broad, right-skewed (with the absorbance tail at longer wavelengths), which can result from the size distributions of nanoparticles presented on Figure 3. The intensity of plasmon resonance depends on the size of the aggregated particles and thus the relation between the number of particles and absorbance intensity is nonlinear. The mean nanoparticle size, electrokinetic potential, and shape are summarised in Table 1.

### 2.2. Evaluation of Bactericidal and Fungicidal Properties of Nanosilver

The evaluation of the disinfectant properties of nanosilver preparations, differing in particle size and morphology, was carried out in two stages. In the first stage, the suspension-test tube method was used, in which three preparations with the best bactericidal and fungicidal properties were selected. During the second stage, the evaluations were performed using the dilution-neutralisation method, according to PN-EN1040 and PN-EN1275. On the basis of the obtained results, the preparation with the best bactericidal and fungicidal properties was selected.

Five strains of microorganisms obtained from the American Type Culture Collection (ATCC) were used in this study:* Escherichia coli *25922,* Pseudomonas aeruginosa* ATCC 27853,* Salmonella enteritidis* ATCC 13076,* Staphylococcus aureus* ATCC 25923, and* Candida albicans* ATCC 10231.

#### 2.2.1. Suspension-Test Tube Method

A tested bacteria suspension of 0.5 on the McFarland scale was diluted in sterile distilled water in order to obtain *Z* ^−1^, *Z* ^−2^, and *Z* ^−3^ suspension dilutions. Then, 1.0 cm^3^ of the bacterial suspension from the *Z* ^−2^ dilution was added to each of eight test tubes containing 9.0 cm^3^ of nanosilver preparations (numbered from 1 to 8). The *Z* ^−3^ suspension in distilled water served as the control sample. The contact time between the bacterial suspension and the preparation amounted to 5 and 30 minutes. After that, samples of 0.1 cm^3^ were taken from each test tube and inoculated on the proper agar substrates (MacConkey, TSA, SS, or Sabouraud). After 24 hours of incubation at 37°C, the colonies were counted. The reduction ratio for each preparation and tested strain was calculated in relation to the control sample.

#### 2.2.2. Dilution-Neutralisation Method

Before the experiment, the tested strains of the microorganisms were proliferated on the proper agar substrates for 24 h at a temperature of 37°C. After incubation, the suspensions of microorganisms from the obtained cultures were prepared in sterile distilled water, in order to achieve the concentration of cells ranging from about 1.5 × 10^8^ to 5 × 10^8^ cfu/cm^3^ for bacteria and 1.5 × 10^6^ cfu/cm^3^ for* C. albicans*. A series of decimal dilutions was prepared from the obtained suspension, and then 0.1 cm^3^ of the suspension of microorganisms from the last four dilutions was inoculated on two plates with the proper agar medium to precisely determine the number of cells in the initial suspension. At the same time, the neutraliser solution was prepared by adding 1.0 cm^3^ of sterile distilled water to 8.0 cm^3^ of neutraliser. The suspension of cells from the *Z* ^−1^ dilution in the amount of 1.0 cm^3^ was transferred to three test tubes containing 9.0 cm^3^ of the examined suspension of silver nanoparticles. After the specified contact time had passed, the action of the studied preparation was neutralised by transferring 1.0 cm^3^ of the bacteria suspension in the preparation to 9.0 cm^3^ of a previously prepared mixture of neutraliser and distilled water. After 5 minutes of neutralisation, culture was performed. 0.1 cm^3^ of the suspension from the neutraliser was transferred to two Petri plates with the proper agar medium. The experiment was performed for 5, 15, 30, and 60 minutes of contact between microorganisms and the nanosilver preparations. The validation of the neutraliser solution was performed by making four mixtures: 8.0 cm^3^ of neutraliser, 1.0 cm^3^ of sterile distilled water, and 1.0 cm^3^ of the suspension of the examined microorganism from the 10^−5^ dilution, while the remaining three each contained 8.0 cm^3^ of neutraliser, 1.0 cm^3^ of the studied preparation, and 1.0 cm^3^ of the suspension of the examined microorganism from the 10^−5^ dilution. Cultures of two plates with the proper agar medium were performed from each test tube (0.1 cm^3^). All the plates were incubated at 37°C for 24 h. After that, all the colonies that had formed on the media, which ranged in number from 15 to 300, were counted in order to evaluate the amount of viable bacteria [cfu]. The following formulas were used in the calculations:

(i) Test bacteria suspension (*N*):
  (1) $$\begin{array}{c} N=\frac{c}{\left(n_{1}+0.1n_{2}\right)\cdot d}, \end{array}$$
 where *c* is the sum of the colonies on all the plates included in the calculation, *n*_1_ is the number of plates included in the calculation from the first dilution, *n*_2_ is the number of plates included from the second dilution, and *d* is dilution factor which corresponds to the first dilution included in the calculation.
(ii) The number of viable bacteria in (Na):
  (2) $$\begin{array}{c} Na=\frac{c}{n\cdot d\cdot V}, \end{array}$$
 where *c* is the sum of colonies counted on both plates, *n* is the number of plates included in the calculation, *d* is dilution factor, and *V* is sample volume.
(iii) Degree of reduction of viable bacteria (*X*):
  (3) $$\begin{array}{c} X=\frac{N\cdot 10^{-1}}{Na}. \end{array}$$

### 2.3. Evaluation of the Disinfection Efficacy of Hatchers Using Nanosilver

Eggs coming from a Polish breed of hens called green legged partridge hens were selected for the study. The experimental group (Group D) consisted of hen eggs disinfected with nanosilver before putting them in the incubator (Incubator D). The control group (Group K) consisted of eggs disinfected using UV irradiation for 30 minutes before putting them in the incubator (Incubator K). In the experimental group, the hatcher and the eggs were fogged with a nanosilver preparation. In both groups, the disinfection efficacy was evaluated based on microbiological analyses of the egg surfaces and the hatchers.

The analyses were performed before (series I) and 30 minutes after disinfection (series II). During the incubation, experiments were carried out three times, that is, after the 1st (series III) and 7th day, on the day of candling the eggs (series IV), and on the 17th day, before the onset of hatch (series V).

The microbiological analysis of the egg surfaces and the hatchers is comprised of the determination of the total bacteria count, total* Staphylococcus* count, and total fungal count.

The eggs/embryos were collected in sterile containers and covered with 50.0 cm^3^ of sterile distilled water with Tween 80. After 15 minutes of shaking, cultures were performed from the obtained washings by the surface plating technique on proper media (Table 2) and incubated for 24 hours at 37°C (bacteria) and for 5 days at 25°C (fungi). After the incubation, the number of microorganisms on the egg surface [cfu/egg] was counted, based on the number of colonies.

Smears from the incubators' walls were collected from an area of 10.0 cm^2^ using a sterile template and sterile swabs (Copan, Italy). After collection, smears were placed in transport tubes containing 20.0 cm^3^ of saline. The collected samples were shaken for 2 minutes. From the obtained suspension, cultures were performed on proper media (Table 2) and incubated for 24 hours at 37°C (bacteria) and for 5 days at 25°C (fungi). After the incubation, the number of microorganisms on the incubator wall [cfu/10 cm^2^] was counted, based on the number of colonies.

### 2.4. The Influence of Nanoparticles on the Concentration of Gaseous Contaminants Generated during Incubation

The evaluation of the gas composition of the disinfected hatchers was performed in parallel with the microbiological analyses.

Air samples were collected from the experimental apparatus (NANO Incubator) and control apparatus (UV Incubator) before and 30 minutes after disinfection. During the incubation, the experiments were carried out three times, that is, after the 1st (series III) and 7th day, on the day of candling the eggs (series IV), and on the 17th day, before the onset of hatch (series V). The concentrations of volatile organic compounds (VOCs) and inorganic compounds, particularly sulphur compounds with strong odorogenous properties, were determined in the collected air samples.

Organic gaseous air contaminants were determined by gas chromatography. Air samples were collected with an electric pump into 2-3-litre Tedlar bags (Sensidyne Inc., Clearwater, USA). The organic compounds contained in the air samples were condensed and then desorbed using a thermal desorption unit (TDV; Model 890, Dynatherm, Analytical Instruments Inc., Oxford, USA) to the gas chromatography system (HP 5890 series II, Hewlett Packard, Santa Clara, USA) equipped with a selective flame photometric detector (FPD) combined with an S-filter with a 393 nm wavelength.

Determination of the inorganic compound content in samples sucked into bubbling washers was performed by ion chromatography with the use of a liquid chromatograph (Waters) connected to an analytical column (IC-PAK Anion HR, Waters Corp., Milord, USA) associated with a conductivity detector and UV Indicator.

## 3. Results and Discussion

### 3.1. Biocidal Properties of Nanosilver

On the basis of microbiological analyses (Table 3) conducted using the test tube method, the three most effective nanosilver preparations, numbers 6, 7, and 8, were chosen. The mentioned preparations reached a high degree of reduction after only 5 minutes of contact (Table 4). The suspensions containing particles of about 10 nm in size, which were stabilised with sodium pyrophosphate, demonstrated the strongest bactericidal and fungicidal activity. The crucial feature affecting the efficacy of the studied preparations was the size of silver nanoparticles and the compounds used to stabilise the nanometric structure. Sodium phosphates dissociate in an aqueous environment, resulting in the formation of phosphate anions which adsorb to the surface of metallic particles and prevent their agglomeration by giving a surface charge to stabilised particles. The result is a high negative electrokinetic potential.

In the other preparations, a similar degree of reduction was reached only after prolonging the contact time to 30 minutes (Table 5). Preparation number 1, containing smaller nanosilver particles (8 nm), demonstrated weaker biocidal activity against* Pseudomonas aeruginosa*,* Escherichia coli*, and* Staphylococcus aureus* than preparations 6, 7, and 8. This could be the result of the positive electrokinetic potential and thus a weaker influence on cell membranes of the tested microorganisms, as well as strong steric stabilisation achieved due to gelatine application and, as a consequence, restricted possibility of the direct action of silver particles. Gelatine is a protein and, at elevated temperatures, its collagen bonds break. A colloidal system then forms, where the stabilisation of metallic particles is facilitated. At that time, spherical stabilisation occurs; that is, the protein chains, due to their complex structure, are an effective spatial factor which prevents the agglomeration of metal particles by surface adsorption.

It was found that the preparations containing silver particles of about 50 nm in size, which were stabilised with gelatine (preparations 2 and 3), showed the weakest antimicrobial effect.* S. aureus* was the most resistant bacteria to all of the tested preparations among the tested microorganisms, whereas the yeast* C. albicans* turned out to be the most sensitive.

The results of the evaluation of the bactericidal and fungicidal properties of nanosilver preparations 6, 7, and 8, which was conducted using the dilution-neutralisation method (PN-EN 1040 and PN-EN 1275), are summarised in Tables 6–8. According to the requirements contained in the abovementioned standards, a preparation meets the requirements of basic bactericidal and fungicidal action when it causes a 10^5^-fold reduction in viable bacteria and 10^4^-fold or greater reduction in the case of fungi, under specific test conditions, in 60 minutes or less. Based on the presented results, it can be concluded that all the preparations achieved the required level of reduction for the majority of the studied strains after only 60 minutes of contact. After 60 minutes of contact, the studied preparations achieved the 10^5^-fold result of reduction for the majority of strains. This situation did not only refer to the Gram-positive bacterium* S. aureus* which, in the case of the evaluation using the test tube method, proved to be the most resistant to the action of silver nanoparticles. Suspension number 6 achieved the required level of reduction after only 15 minutes in case of the yeast* C. albicans *(Table 6). A similar result for the same strain was obtained in the case of preparation number 7 (Table 7), but the strongest biocidal action was displayed by suspension number 8 (Table 8). After 30 minutes of contact, the desired level of reduction was noted for* C. albicans, P. aeruginosa*, and* S. enteritidis*. The two remaining preparations (6 and 7) showed disinfecting action in this period of time only towards* S. enteritidis*. On the basis of these results, preparation number 8 was selected for application tests.

### 3.2. Application Tests

In this part of the study, significant microbiological contamination of the eggshells of eggs designed for incubation (Table 9) was observed. On the surface of the eggs, the total bacteria count amounted to, on average, 9.8 × 10^4^ cfu/egg, while the average fungal count was 2.1 × 10^2^ cfu/egg. Therefore, maintaining microbiological purity is possible with properly conducted disinfection of eggs and hatchers. In these tests, the effectiveness of the studied nanosilver preparation in the case of the total bacteria count was on a level similar to the effectiveness of UV disinfection (Table 9). After 30 minutes, the number of bacteria on control eggs and on eggs disinfected with silver nanoparticles amounted to ×10^4^, while on the second day of the incubation it was even larger on the eggs treated with the preparation. Visible changes in the total bacteria count were observed on the 7th day of incubation, when the mean number of bacteria on control eggs was almost six times greater compared to experimental eggs. On the 17th day of incubation, a significant increase in the mean number of bacteria on the surface of all eggs was observed, compared to the earlier stages of incubation. The cause of this situation was probably secondary contamination as a result of candling the eggs on the 7th day. The average difference between the amount of bacteria on control eggs and tested eggs varied and was half of the amount on experimental eggs.

In the case of the total* Staphylococcus* count, the contamination remained on a level similar to the total bacteria count (Table 9). No differences in the mean number of staphylococci were observed between control and experimental eggs up to the 17th day of incubation, when the presence of cocci was not detected on experimental embryos.

Total fungal counts remained at a stable level on control and experimental eggs (Table 9). On the 17th day of incubation, in both groups, the presence of fungi was not observed.

These results show that the disinfecting action of nanosilver towards all the studied groups of microorganisms was observed from the 7th day of incubation. An increase in the total microorganism count on the 7th day of incubation could be connected with secondary contamination of the surface of the eggs as a result of candling during incubation.

Before disinfection, differences were detected in the contamination level of the incubators. The experimental incubator was more severely bacteriologically contaminated compared to the control incubator (Table 10). After 30 minutes of disinfection and putting the eggs in the incubator, the contamination of the control incubator increased, certainly as a result of secondary contamination, whereas the contamination of the experimental incubator was significantly decreased. On the 2nd day of incubation, the mean bacterial contamination decreased in both the control and experimental incubators, but the mean number of bacteria in the experimental incubator was significantly lower than in the control one. On the 17th day, the mean total bacteria count per cm^2^ of the control incubator decreased, as did the number of bacteria in the experimental incubator.

The total* Staphylococcus* count was low and fluctuated, which could be a result of secondary contamination from the air (Table 10).

The total fungal count displayed a decreasing tendency throughout the incubation period. Significant differences between the control and the experimental incubator continued from the onset of incubation up to the 17th day.

On the basis of the mean total bacteria count and total fungal count, it can be stated that the studied nanosilver preparation demonstrated good bactericidal and fungicidal effectiveness on the surface of the hatchers. The fluctuations in the number of staphylococci can be explained by secondary contamination and may not be connected with the action of nanosilver.

**Figure 4:**
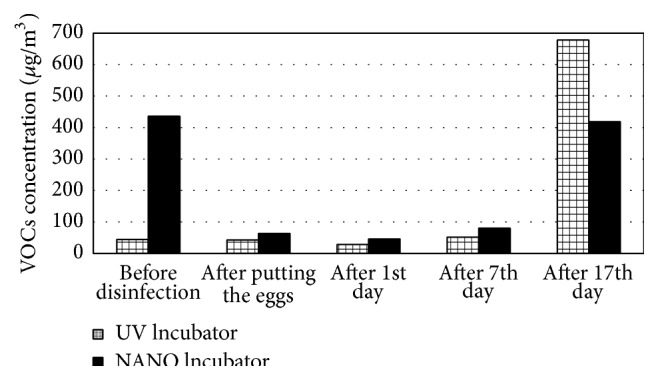
VOC concentrations in the air inside the incubators during the incubation [*μ*g/m^3^].

**Figure 5:**
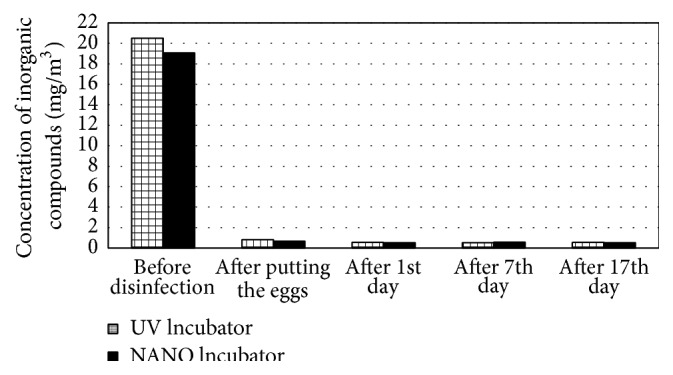
Concentration of inorganic gaseous compounds in the air inside the incubators during the incubation [mg/m^3^].

**Figure 6:**
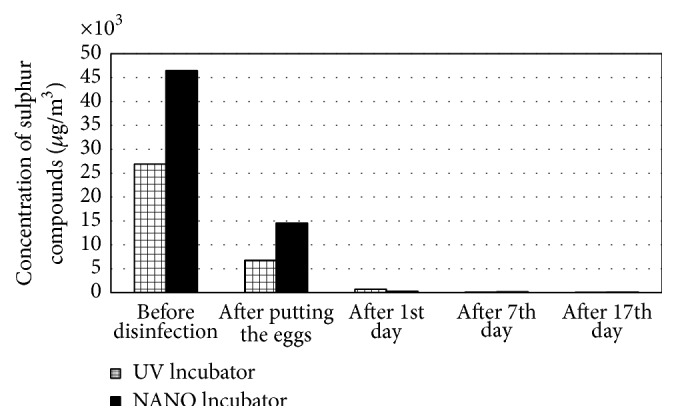
Concentration of sulphur compounds in the air during the incubation [*μ*g/m^3^].

Very good results were achieved in the case of organic gaseous contaminants. After the application of the nanosilver preparation, these levels decreased by 86% (Figure 4). During the 17-day incubation, an increase in the VOC concentration was observed in both apparatuses. At the end of the process, the level of contaminants in the air inside the UV Incubator was 40% higher than in the NANO Incubator. The chromatographic studies did not demonstrate the accumulation of inorganic compounds and sulphur compounds in the apparatuses (Figures 5 and 6). In the last series of studies, the concentration of sulphur compounds in the air inside the UV and NANO Incubators was determined to be 11.0 *μ*g/m^3^ and 14.9 *μ*g/m^3^, respectively.

Among the identified VOC compounds, the highest concentration in the UV Incubator was seen for 2-pentanamine (284.3 *μ*g/m^3^) and cyclobutanol (247.1 *μ*g/m^3^), whereas, in the NANO Incubator, the highest concentration was seen for hexanal (255.4 *μ*g/m^3^). In the apparatus disinfected with nanosilver, the presence of 2-pentanamine, 2-methylpentane, 2-methyl-1-propanol, trichloroethylene, and toluene (Table 11) as well as sulphur compounds such as carbonyl sulphide (COS) and methyl mercaptan (Table 12) was not detected. The applied method of disinfection did not have an influence on the level of the determined inorganic contaminants (Table 13).

## 4. Conclusion

According to current standards of production, in the majority of food industry plants, including the egg-poultry industry, the obligatory control of microbiological and chemical quality of air and production surfaces has been introduced, which means an obligation to ensure proper cleanliness. In chick hatcheries, the hatchers constitute a critical point because thermal and humidity conditions as well as the presence of biological material inside them pose a significant microbiological hazard. In such places, not without reason, great emphasis has been placed on maintaining microbiological purity, which is achieved only by properly conducted disinfection of hatchers and eggs.

The present research demonstrated that the nanosilver preparation applied for the disinfection of eggs and hatchers reduced microbiological contamination. The preparation used showed bactericidal and fungicidal effectiveness comparable to UV radiation, and its effectiveness increased throughout the incubation. In the case of fungi, on the 7th day of incubation, fogging with nanosilver gave better protection to the surface of eggs than irradiation with UV.

Very good results were achieved in the case of organic gaseous contaminants. After application of the nanosilver preparation, these levels decreased by 86%. The level of contaminants in the air inside the incubator decontaminated with UV was 40% higher than in the incubator disinfected with nanosilver.

Proper egg incubation conditions during hatching are very important and result in effective hatching and healthy chicks. Intensively running metabolic processes and embryo respiration require that the air inside incubator contains a sufficient amount of oxygen. Gases released during the incubation have to be removed from the apparatus, because excessive amounts can lead to poisoning and embryo decay, so proper air exchange is of great importance. The application of nanosilver may be used to optimise the process of incubation. As this study shows, nanosilver is not able to replace efficiently functioning ventilation but may be an element supporting the elimination of gaseous contaminants.

## Figures and Tables

**Table 1:** The parameters of the processes of obtaining silver nanoparticles and the properties of the obtained products.

Number of the preparation	Stabiliser	Reducer	Concentration of Ag^+^ ions [mg/dm^3^]	Molar ratio stabiliser: Ag^+^	Gelatine weight [g]	Molar ratio reducer: Ag^+^	pH	Temperature of the process [°C]	Duration of the process [min]	Potential *ζ* [mV]	*d*_av_ [nm]	Shape of the particles
1	Gelatine	L(+) ascorbic acid	50	—	0.2	3.0	4.61	130	30	10.4	8.2	Oval
2	Gelatine	L(+) ascorbic acid	100	—	0.2	5.5	4.38	130	15	14.1	48.7	Oval
3	Gelatine	L(+) ascorbic acid	200	—	0.2	5.5	4.38	130	15	14.4	50.0	Oval
4	TPPS	L(+) ascorbic acid	50	5.0	—	2.0	9.80	150	30	−29.8	49.3	Oval
5	TPFS	L(+) ascorbic acid	50	5.0	—	5.0	11,00	110	30	−31.1	51.3	Oval
6	Sodium pyrophosphate	Glucose	50	7.0	—	6.0	8.00	110	30	−37.5	11.5	Oval
7	Sodium pyrophosphate	Glucose	100	5.0	—	4.0	7.10	130	15	−30.7	9.6	Oval
8	Sodium pyrophosphate	Glucose	50	5.0	—	4.0	7.00	110	15	−31.5	10.2	Polyhedral

**Table 2:** Microbiological media used for the microbiological evaluation of eggs and hatchers.

Determination	Microbiological medium
Total bacteria count	Enriched agar
Total *Staphylococcus* count	Chapman medium
Total fungi count	Sabouraud medium

**Table 3:** Percentage reduction in the amount of microorganisms compared to the control sample for the tested nanosilver preparations.

Tested strain	Preparation number															
	1		2		3		4		5		6		7		8	
	Contact time [min]															
	5	30	5	30	5	30	5	30	5	30	5	30	5	30	5	30
*C. albicans *ATCC	100	100	15	52	24	54	83	100	55	100	100	100	100	100	100	100
*S. enteritidis* ATCC	33	100	18	91	58	100	19	100	39	100	99	100	100	100	92	100
*P. aeruginosa* ATCC	86	94	74	44	79	54	70	98	67	95	93	100	99	99	99	100
*S. aureus* ATCC	40	60	1	5	70	52	−3	74	−8	56	70	92	82	95	79	93
*E. coli* ATCC	70	97	21	15	33	19	79	97	55	80	99	100	100	100	94	100

**Table 4:** The number of bacterial and fungal cells grown after five minutes of contact with nanosilver compared to the control group.

	*Candida albicans* ATCC		*Salmonella enteritidis* ATCC		*Pseudomonas aeruginosa* ATCC		*Staphylococcus aureus* ATCC		*Escherichia coli* ATCC	
	×10^5^ cfu		×10^5^ cfu		×10^6^ cfu		×10^6^ cfu		×10^5^ cfu	
	M	SD	M	SD	M	SD	M	SD	M	SD
Control	28.9	2.1	180.5	127.5	187.8	15.9	78.6	30.9	91.3	26.3
Preparation										
1	0	0	54.3	5.9	26	17.8	44.1	13.3	28.3	15.7
2	24.3	2.6	87.5	74	152.5	10.6	70.5	14.7	70	14
3	21.8	5.9	54	10.4	121	5.7	25	29.7	58.3	12.4
4	4.7	5.2	99.9	33.1	55.8	10	69.9	12.3	18.5	10.4
5	12.7	5	82.2	6.5	62.8	20.2	75.3	15.5	39.5	14.6
6	0	0	0.7	0.8	13.1	15.7	21.9	17.3	0.8	1.3
7	0	0	0.3	0.6	2.3	1.4	13.1	22.2	0	0
8	0	0	8.7	7.5	1.2	0.9	19.4	26.9	4.2	6.5

**Table 5:** The number of bacterial and fungal cells grown after 30 minutes of contact with nanosilver compared to control group.

	*Candida albicans* ATCC		*Salmonella enteritidis* ATCC		*Pseudomonas aeruginosa* ATCC		*Staphylococcus aureus* ATCC		*Escherichia coli* ATCC	
	×10^5^ cfu		×10^5^ cfu		×10^6^ cfu		×10^6^ cfu		×10^5^ cfu	
	M	SD	M	SD	M	SD	M	SD	M	SD
Control	29.8	3.7	261.9	225.7	109.9	43.6	85.7	35.9	50.3	17.9
Preparation										
1	0	0	0	5.4	8.2	25.8	19.8	1.2	2	15.7
2	9.7	12.4	7.3	60.6	22.2	67.9	16.5	41.2	11.3	14
3	10.8	0.1	0.1	48.1	14	38.1	41.7	38	8.7	12.4
4	0	0	0	1.9	1.3	16.9	10.3	1.3	1.4	10.4
5	0	0	0	4.7	2.9	29.2	19.7	8.5	5.4	14.6
6	0	0	0	0	0	7.3	14.9	0	0	1.3
7	0	0	0	0.5	1.2	3.4	3.1	0	0	0
8	0	0	0	0	0	6.4	8.8	0	0	6.5

**Table 6:** Results of the evaluation using the dilution-neutralisation method for suspension number 6.

Tested organism	Bacterial and fungal suspension tested (*N*)	The number of viable bacteria and fungi (cfu/cm^3^) in the studied mixture (Na) in time			
		5 min	15 min	30 min	60 min
*Escherichia coli *ATCC	9.9 × 10^6^	—	2.6 × 10^4^	1.9 × 10^3^	0.0
*Pseudomonas aeruginosa *ATCC	2.75 × 10^8^	—	7.4 × 10^4^	2.0 × 10^3^	1.0 × 10^2^
*Salmonella enteritidis *ATCC	2.05 × 10^7^	9.6 × 10^4^	1.8 × 10^3^	0.0	0.0
*Staphylococcus aureus* ATCC	1.84 × 10^8^	—	2.6 × 10^5^	2.0 × 10^5^	5.3 × 10^4^
*Candida albicans *ATCC	9.31 × 10^5^	3.6 × 10^3^	0.0	0.0	0.0

Reduction of viable bacteria and fungus at the studied time					
*Escherichia coli *ATCC		—	3.8 × 10^1^	5.4 × 10^2^	>10^5^
*Pseudomonas aeruginosa *ATCC		—	3.7 × 10^2^	1.4 × 10^4^	2.8 × 10^5^
*Salmonella enteritidis *ATCC		2.1 × 10^1^	1.2 × 10^3^	>10^5^	>10^5^
*Staphylococcus aureus* ATCC		—	7.1 × 10^1^	9.2 × 10^1^	3.5 × 10^2^
*Candida albicans *ATCC		2.6 × 10^1^	>10^5^	>10^5^	>10^5^

**Table 7:** Results of the evaluation using the dilution-neutralisation method for suspension number 7.

Tested organism	Bacterial and fungal suspension tested (*N*)	The number of viable bacteria and fungi (cfu/cm) in the studied mixture (Na) in time			
		5 min	15 min	30 min	60 min
*Escherichia coli *ATCC	9.9 × 10^6^	—	2.2 × 10^4^	5.5 × 10^2^	0.0
*Pseudomonas aeruginosa *ATCC	2.75 × 10^8^	—	3.5 × 10^4^	1.0 × 10^2^	0.0
*Salmonella enteritidis *ATCC	2.05 × 10^7^	1.1 × 10^5^	1.0 × 10^2^	0.0	0.0
*Staphylococcus aureus* ATCC	1.84 × 10^8^	—	1.8 × 10^5^	1.1 × 10^5^	7.0 × 10^3^
*Candida albicans *ATCC	9.31 × 10^5^	2.0 × 10^3^	2.0 × 10^2^	0.0	0.0

Reduction of viable bacteria and fungus at the studied time					
*Escherichia coli *ATCC		—	4.5 × 10^1^	1.8 × 10^3^	>10^5^
*Pseudomonas aeruginosa *ATCC		—	8.0 × 10^2^	2.8 × 10^5^	>10^5^
*Salmonella enteritidis *ATCC		2.0 × 10^1^	2.1 × 10^4^	>10^5^	>10^5^
*Staphylococcus aureus* ATCC		—	1.0 × 10^4^	1.7 × 10^2^	2.6 × 10^3^
*Candida albicans *ATCC		4.7 × 10^1^	4.7 × 10^2^	>10^5^	>10^5^

**Table 8:** Results of the evaluation using the dilution-neutralisation method for suspension number 8.

Tested organism	Bacterial and fungal suspension tested (*N*)	The number of viable bacteria and fungi (cfu/cm^3^) in the studied mixture (Na) in time			
		5 min	15 min	30 min	60 min
*Escherichia coli *ATCC	9.9 × 10^6^	—	2.1 × 10^4^	5.0 × 10^0^	0.0
*Pseudomonas aeruginosa *ATCC	2.75 × 10^8^	—	5.4 × 10^4^	1.0 × 10^3^	0.0
*Salmonella enteritidis *ATCC	2.05 × 10^7^	1.2 × 10^5^	4.5 × 10^2^	0.0	0.0
*Staphylococcus aureus* ATCC	1.84 × 10^8^	—	3.3 × 10^5^	2.0 × 10^5^	6.9 × 10^4^
*Candida albicans *ATCC	9.31 × 10^5^	2.3 × 10^3^	0.0	0.0	0.0

Reduction of viable bacteria and fungus at the studied time					
*Escherichia coli *ATCC		—	4.8 × 10^1^	2.0 × 10^5^	>10^5^
*Pseudomonas aeruginosa *ATCC		—	5.1 × 10^2^	2.8 × 10^4^	>10^5^
*Salmonella enteritidis *ATCC		1.7 × 10^1^	4.6 × 10^3^	>10^5^	>10^5^
*Staphylococcus aureus* ATCC		—	5.5 × 10^1^	9.3 × 10^1^	2.7 × 10^2^
*Candida albicans *ATCC		4.1 × 10^1^	>10^5^	>10^5^	>10^5^

**Table 9:** Microbiological contamination of eggs during incubation [cfu/egg].

Total count of	Group	Before disinfection	30 min after disinfection	2nd day of incubation	7th day of incubation	17th day of incubation
Bacteria	K	9.8 × 10^4^	2.3 × 10^4^	2.1 × 10^3^	3.5 × 10^3^	4.5 × 10^4^
	D	9.8 × 10^4^	2.5 × 10^4^	6.9 × 10^3^	6.0 × 10^2^	2.7 × 10^4^

*Staphylococcus*	K	1.8 × 10^5^	3.3 × 10^4^	2.1 × 10^3^	3.3 × 10^3^	1.3 × 10^3^
	D	1.8 × 10^5^	3.6 × 10^4^	6.2 × 10^3^	3.3 × 10^2^	0

Fungi	K	2.1 × 10^2^	1.0 × 10^2^	1.3 × 10^2^	7.0 × 10^2^	0
	D	2.1 × 10^2^	1.0 × 10^2^	1.3 × 10^2^	1.0 × 10^2^	0

**Table 10:** Microbiological contamination on the walls of the hatchers [cfu/cm^2^].

Total count of	Incubator	Before disinfection	30 min after disinfection	2nd day of incubation	7th day of incubation	17th day of incubation
Bacteria	K	36.7	463.30	61.7	20.0	13.3
	D	516.7	3.3	5.0	10.0	13.3

*Staphylococcus*	K	0.0	0.0	3.3	0.0	3.3
	D	3.3	0.0	0.0	0.0	8.3

Fungi	K	20.0	30.0	31.7	13.3	0.0
	D	15.0	16.7	8.3	1.7	0.0

**Table 11:** VOC concentrations in the air inside the incubators on the 17th day of incubation [*µ*g/m^3^].

Compound	Incubator	
	UV	NANO
Total	677.0	415.4
Methane	1.5	3.2
Propanol	4.0	2.1
Cyclobutanol	247.1	10.1
1-Butanol	21.6	8.9
2-Pentanamine	284.3	—
2-Methylpentane	18.9	—
2-Methyl-1-propanol	1.6	—
Benzene	13.3	13.5
Trichloroethylene	7.5	—
1-Pentanol	1.9	14.2
Indole	15.6	54.3
Ethylbenzene	1.2	1.9
Xylenes	53.3	34.7
Phenol	3.0	2.9
Methylcyclopentane	—	14.2
Toluene	2.0	—
Hexanal	0.5	255.4

**Table 12:** Concentration of sulphur compounds in the air inside the incubators on the 17th day of incubation [*µ*g/m^3^].

Compound	Incubator	
	UV	NANO
Total	11.0	14.9
Diethyl sulphide	1.9	1.6
Dipropyl sulphide	—	0.4
Methylpropyl sulphide	1.9	3.1
Carbonyl sulphide	0.1	—
Methyl mercaptan	0.7	—
Ethyl mercaptan	—	1.4
Isopropyl mercaptan	0.9	0.9

**Table 13:** Concentration of inorganic gaseous compounds in the air inside the incubators on the 17th day of incubation [mg/m^3^].

Compound	Incubator	
	UV	NANO
Total	0.5	0.5
Ammonia	0.1	0.1
Nitrates	0.1	0.1
Nitrites	0.1	0.1
Chlorides	0.1	0.1
Sulphates	0.1	0.1
